# Extra-Articular Tenosynovial Chondromatosis of the Finger: A Case Series Study of Three Cases, One Including Excessive Osseous Invasion

**DOI:** 10.2174/1874325001711010417

**Published:** 2017-05-17

**Authors:** Akio Sakamoto, Takahiko Naka, Eisuke Shiba, Masanori Hisaoka, Shuichi Matsuda

**Affiliations:** 1 The Department of Orthopaedic Surgery, Graduate School of Medicine, Kyoto University, Kyoto, Japan; 2 Shimosone Clinic of Orthopedics and Osteoporosis, Kitakyushu, Japan.; 3 The Department of Pathology and Oncology, School of Medicine, University of Occupational and Environmental Health, Kitakyushu, Japan.

**Keywords:** Extra-articular, Tenosynovial chondromatosis, Osteochondromatosis, Finger, Hand, Bone

## Abstract

**Background:**

Synovial chondromatosis is characterized by cartilaginous metaplasia in synovial tissues. Extra-articular tenosynovial chondromatosis is considered to be an anatomical counterpart of articular synovial chondromatosis. Extra-articular tenosynovial chondromatosis occurs preferentially in the hand, although its frequency is low.

**Results:**

We report three cases of extra-articular tenosynovial chondromatosis. A 65-year-old female presented with a history of symptoms over 40 years related to the dorsum of her index finger (Case 1), A 46-year-old female presented with a 6-month history of symptoms at the volar surface of her middle finger (Case 2), and a 66-year-old male presented with a 3-month history of symptoms in a dorsal ring finger. Case 2 had evidence of ossification, which could be classified as osteochondromatosis. Interestingly, the index finger lesions (Case 1) were accompanied by excessive bone involvement. The signal intensity of T2-weighted magnetic resonance imaging varies from low to high, possibly reflecting histological variations, such as ossification and fatty tissue changes. All lesions were resected without complications.

**Conclusion:**

Variations in anatomical sites suggest that overuse or mechanical overloading was not causative. Extensive involvement of the nearby tendon and joint capsule, as well as the bone, would require attention during the resection. Preoperative analysis of images is important, not only for the diagnosis, but also to assess the extent of the lesion, particularly given the complex anatomy of the finger.

## INTRODUCTION

Synovial chondromatosis is an unusual condition that is characterized by cartilaginous metaplasia in the synovial membrane of joints, bursae, and tendon sheaths [1-3]. Articular synovial chondromatosis typically involves large joints such as the knee and the hip [4, 5]. Extra-articular tenosynovial chondromatosis is considered to be an anatomical counterpart of articular synovial chondromatosis, and tends to occur in the hands and feet [6-8].

Extra-articular tenosynovial chondromatosis of the finger is a rare condition. Signs and symptoms include swelling, reduced range of motion, and pain [6]. Herein, we report three cases of extra-articular tenosynovial chondromatosis of the finger, using each case to characterize the clinical features of the associated pathology, and we also present radiologic and histologic findings. Emphasis is given to the anatomical specificity of fingers and the complex nature of the structures involved.

## CASES

Clinical data are summarized in Table **1**. Two females and one male patient (65, 46, and 66 years old, respectively, at the time of surgery; mean age 59.0 ± 11.3 years) were among the three cases of extra-articular tenosynovial chondromatosis of the finger. All cases were right-hand dominant. The male patient was retired, and the other 2 female patients were housewives. There was no history of trauma, repetitive stress, and/or over usage of the fingers among the cases. The lesions were located on the dorsal side in two cases, and the volar side in one case. Two of the lesions were located under the tendon, and for the remaining case the lesion was above the tendon. Multiple nodules were observed in two cases, whereas a singular nodule was present in one case. Each nodule was 1 cm to 2 cm in size. Intraosseous invasion was seen in the case with calcification. Adjacent degenerative changes were not observed in any cases. The duration from onset varied; it was 40 years, 6 months, and 3 months in these cases. The lesions were treated by curettage. We describe the details of each case below.

### Case 1

**Fig. (1):**
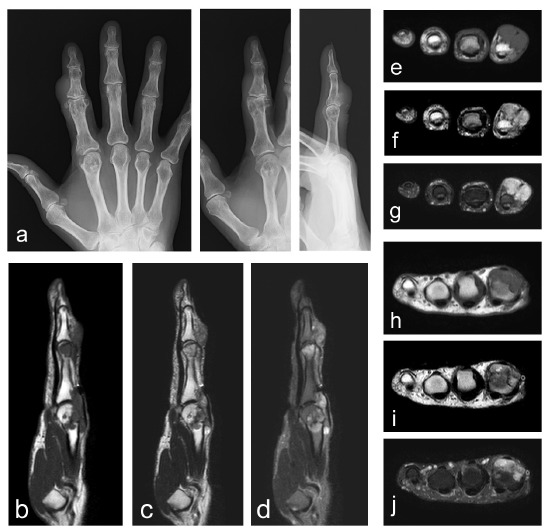
Images of a 65-year-old female with extra-articular tenosynovial chondromatosis. The plain radiographs of the index finger of the right hand show irregularity of the cortex at the distal end of the proximal phalanx and of the second metacarpal bone (a: anteroposterior; left, oblique; middle, lateral; right). MRIs (b-j) show multinodular lesions around the proximal interphalangeal joint (e-g) and metacarpophalangeal joint (h–j) levels with homogeneous low signal intensity on the T1-weighted images (b, e, and h), and heterogeneous low to high signal intensity on the T2-weighted images (c, f, and i) with fat-suppression (d, g and j). Osseous invasion is apparent.

**Fig. (2):**
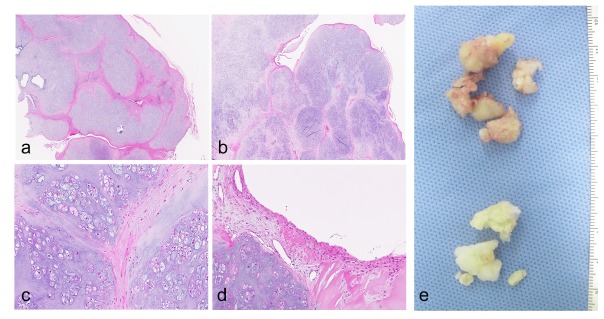
Images of an extra-articular tenosynovial chondromatosis of the index finger in a 65-year-old female (the same patient in Fig. (**1**)). Low-magnification views of H&E stained histology sections showing multiple cartilaginous nodules (a and b). H&E stained histology sections of cartilaginous cells with chondroid stroma (c) and cartilaginous nodules lined by synovium (d). Resected specimens of extraosseous (top) and intraosseous (bottom) origin (e). Original magnifications of panels: a and b, x40; c, x100; d, x200.

A 65-year-old female presented with complaints of a painless but hard irregular swelling on the dorsum of her index finger. She described the swelling as having slightly increased in size over the 40-year period since onset, but had noticed more of an increase over the previous 2 months. There was no history of trauma or overuse. Despite experiencing that a full grip was difficult; we noted a slightly reduced range of motion in the proximal interphalangeal (PIP) joint, no restrictions in her daily activities were reported. Multiple lesions comprising hard masses, some of which had elastic properties, were palpable over the dorsum of the PIP joint and metacarpophalangeal (MP) joint of the index finger. Plain radiographs of the index finger showed evidence of calcification. Irregularity in the adjacent bone surfaces was observed (Fig. **1a**). A magnetic resonance image (MRI) showed multinodular lesions under the extensor tendon with homogeneous intermediate signal intensity on the T1-weighted images and heterogeneous low to high signal intensify on the T2-weighted images. The lesions extended from the PIP joint to the MP joint on the dorsal side. Osseous invasion was evident (Figs. **1b**-**j**). The resection was performed under general anesthesia, with a tourniquet at the upper arm, through a zigzag incision on the dorsum of the index finger, from the PIP joint to the MP joint. The presence of a white colored lesions covered by synovial tissue was confirmed before each resection. At the PIP joint the capsule was connected to a lesion, but the lesions did not extend into the joint. The involved extensor tendon was preserved, although parts of the lateral hood and joint capsule were resected with the lesion. Lesions in the bone at the distal proximal phalanx and the distal metacarpal were curetted. Histologically, the lesion is composed of multiple nodules of hyaline cartilage containing chondrocytes arranged in clusters and separated by fibrous septa. Synovial lining was visible covering the cartilaginous nodules. The features are compatible with synovial chondromatosis (Fig. **2**). Malignant or infectious features were absent. Two years after the operation, a follow-up physical examination confirmed no recurrence of a lesion. A slight reduction in the range of motion in the PIP joint was still evident, but there was no effect on the activities of the daily life.

### Case 2

**Fig. (3):**
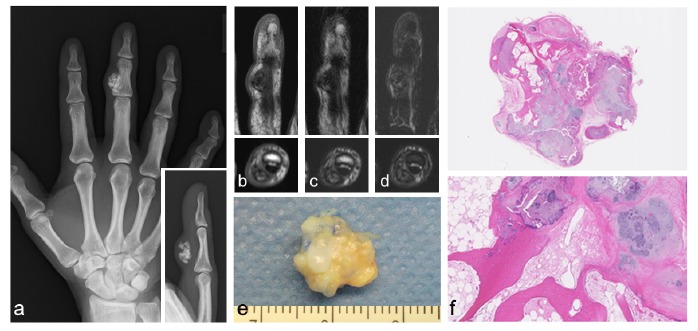
A 46-year-old female with extra-articular tenosynovial chondromatosis of the middle finger. Plain radiographs of the finger show an osseous nodule in the middle finger (a). The lesion has heterogeneous low- to high-signal intensity on T1-weighted (b), T2-weighted (c), and T2-weighted fat-suppression (d) images. A photograph of the resected specimen (e). Images of H&E histology sections of cartilaginous nodules with ossification and bone marrow-like fat tissue (f). Original magnification of panel f: x10 (top) and x40 (bottom).

A 46-year-old female complained of swelling in her right middle index finger that she had endured for 6 months. The range of motion of this finger was slightly reduced in flexion because of the mass of a lesion. There was no history of pain or trauma. The patient reported no tenderness over the area of the lesion. Plain radiographs of the hand showed a small osseous node in the middle finger at the radial flexor side (Fig. **3a**). An MRI revealed a lesion located above the flexor tendon. The signal intensity of the lesion was heterogeneous with low to high signal intensity on the T1- and T2-weighted images. Foci of high signal intensity were also observed in the T1- and T2-weighted images, suggesting the presence of fatty tissue (Figs. **3b**-**d**). A resection was performed under general anesthesia, with a tourniquet applied at the upper arm. A curved incision was made starting at the base of the radial side of the index finger and extending over the lesion at the proximal phalanx. After confirming the presence of a white colored hard lesion, it was dissected from the surrounding tissue. Histologically, the lesion was composed of osteocartilagenous tissue, with irregularly thickened bone trabeculae and fatty marrow. The lesion was surrounded by fibrovascular connective tissue. The features are compatible with synovial chondromatosis (Figs. **3e** and **f**). Malignant or infectious features were absent. No recurrence was observed at the time of follow-up 11 months after the operation. Full range of motion was restored without any effect on activities of daily living.

### Case 3

**Fig. (4):**
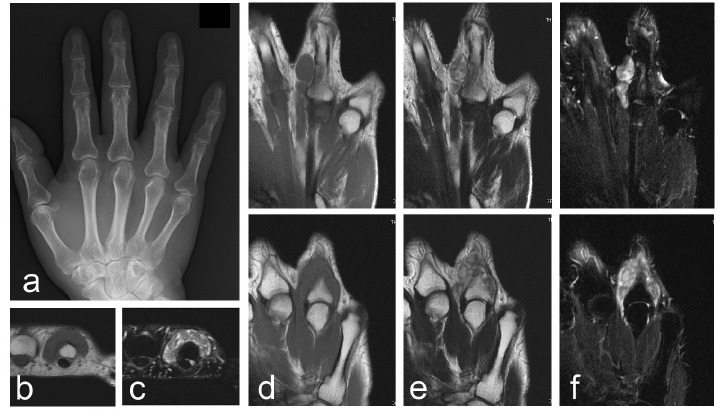
Images of a 66-year-old male with extra-articular tenosynovial chondromatosis of the right ring finger. Plain radiographs of the hand showed no abnormality (a). An MRI showing multinodular lesions with homogeneous low signal intensity on the T1-weighted images (b and d) and heterogeneous low to high signal intensity on the T2-weighted images (e) on the dorsal side of the proximal phalanx. Fat suppression on the T2-weighted images demonstrates an extension of the lesion (c and f). The top and bottom images of panel d-f are sequential.

A 66 year-old male complained of a painless swelling on his right ring finger that he had noticed for 3 months. There was no limitation in the range of motion of the finger, although the lesion caused discomfort in full grip. There was no history of trauma or overuse. On physical examination, the mass of the lesion was palpable at the dorsum of the right finger at the distal of the MP joint. The plain radiographs revealed no apparent abnormalities. No calcification was observed (Fig. **4a**). An MRI showed multinodular lesions located at the dorsal aspect of the proximal phalanx under the tendon. The lesions had homogeneous intermediate signal intensity on the T1-weighted images and heterogeneous low to high signal intensity on the T2-weighted images (Figs. **4b**-**f**). The resection was performed under general anesthesia, with a tourniquet applied at the upper arm, and a mid-dorsal incision was made. Parts of the lateral hood were resected along with the lesion. Histology showed that the lesion was composed of multiple nodular or lobular structures of what appeared to be mature hyaline cartilaginous tissue, surrounded by synovial tissue (not shown in figure), the features of which were compatible with synovial chondromatosis. No signs of malignancy or infection were observed. A follow-up performed 5 years and 2 months after surgery confirmed no recurrence of a lesion. Full range of motion was maintained without any complications.

## DISCUSSION

The condition of synovial chondromatosis is thought to be due to metaplasia [2-4]. Extra-articular tenosynovial chondromatosis in the hand has been reported to commonly affect adults in the fifth decade of life [6]. The mean age of patients at the time of surgery was 59.0 ± 11.3 years. However, the 65-year-old patient who had a 40-year history of extra-articular tenosynovial chondromatosis suggests that the initial symptoms can occur at approximately 20 years old, or at any adult age.

Extra-articular tenosynovial chondromatosis more frequently involves the flexor surfaces of fingers [2, 7]. In the current series, the anatomical location of the finger varied. Moreover, no specific determinant such as trauma was identified. The absence of significant trauma to the affected area was consistent with a previous report [2]. These findings, together with the variation in the anatomical locations, suggest that extra-articular tenosynovial chondromatosis in the finger may not be associated with overuse or mechanical overloading.

Histologically, synovial chondromatosis is characterized by nodular hyaline cartilage surrounded by a synovial lining [2, 5]. Some nodules may have bony trabeculae with bone marrow, and be referred to as osteochondromatosis, as seen in case 1 in this report. The differential diagnoses of extra-articular tenosynovial chondromatosis include cartilage forming benign tumors of a soft-tissue chondroma and a periosteal chondroma [6]. A cartilage-forming benign tumor is known for its lack of synovial lining. Moreover, multiple nodules are not likely with these neoplastic lesions.

The plain radiographs of extra-articular tenosynovial chondromatosis in these cases revealed the existence of calcification or ossification in the cartilaginous nodules [2, 4]. Erosion of the bone surface is also a possible finding on plain radiographs [5, 6]. In the case of the 65-year-old patient, irregularity of the cortical bone adjacent to the lesions was observed, due to the osseous invasion of the lesion. Complications of articular synovial chondromatosis include secondary degenerative osteoarthritis in the knee or hip [2]. However, in cases of extra-articular tenosynovial chondromatosis, adjacent joints are reported to be normal [5], as was seen in the current cases. Extra-articular tenosynovial chondromatosis is not associated with the nearby joint and thus is referred to as “extra-articular” [6-8].

MRIs can clearly reveal the location of the lesion [9]. The signal intensity varied by case. Heterogeneous signal intensities appeared to reflect the histological variation in lesions that contained chondroid material, calcification, and ossification, as well as fatty tissue changes in bone marrow [2]. The appearance of synovial chondromatosis in MRIs is characterized as lobulated, homogeneous, and with intermediate intra-articular signal intensity, similar to that of muscle in T1-weighted images, with high signal intensity on T2-weighted images. Focal areas of low signal intensity with all pulse sequences corresponds to regions of calcification or ossification, and high-signal-intensity foci are relative to fatty tissue [9].

Malignant transformations in which synovial chondromatosis progressed to chondrosarcoma have been reported. The rate of incidence in one study was four out of 155 cases of synovial chondromatosis (approximately 2.5%) [10]. Of these four cases, three involved the hip joint and one case involved the elbow joint [10]. In cases of chondromatosis of the finger, there is one report of synovial chondrosarcoma involving the MP joint of the thumb in a 69-year-old man [11]. However, the lack of histological findings characteristic of synovial chondromatosis failed to support the progression of synovial chondromatosis to a malignancy [11].

For articular synovial chondromatosis in the hip and knee, although the risk of malignant transformation is small, resection of the cartilaginous lesions and synovectomy tend to be recommended [10]. In contrast, a treatment indication for articular synovial chondromatosis in upper extremities has not been established [12], although surgical resection seems to be a preferred option for extra-articular tenosynovial chondromatosis [5, 6, 13]. A specific indication for resection is less likely given that malignant transformations are not known to be common and a nonsurgical approach involving monitoring would depend largely on a patient’s preference when the signs and symptoms of swelling, reduced range of motion, and pain are moderate. In the current three cases, the signs and symptoms were not severe, but resections were recommended even though the diagnoses were benign neoplastic lesions, because of a prevailing fear of enlargement of the lesions. The extensive nature of extra-articular tenosynovial chondromatosis in the finger, with tendon and/or bone involvement, supports resection as a plausible option to manage the condition. In the case of articular synovial chondromatosis, resection is typically performed via either an open or arthroscopic approach [12], while as in the case of extra-articular synovial chondromatosis, open resection is performed.

In the current series, the recurrence of lesions was not observed during the follow-up periods. The overall recurrence rate for articular chondromatosis ranges from 3% to 23% [5, 7]. In cases of extra-articular tenosynovial chondromatosis in the digits, the frequency of local recurrence has been reported be high; that is, 10 out of 19 cases have been known to recur based on the reports that we found [6, 8].

## CONCLUSION

This case series study reports three cases of extra-articular tenosynovial chondromatosis of the fingers, including one case with excessive bone involvement. There were no specific determinants for the onset of the pathology identified among these cases. The variation in anatomical site suggests the cause was not associated with overuse or mechanical overload. The MRI findings reflect the histological variation among cases. Extensive lesions with tendon or bone involvement add to the anatomical complexity, making preoperative image analysis imperative, not only for the diagnosis, but also when planning the resection.

## Figures and Tables

**Table 1:** Clinical summary of cases of extra-articular tenosynovial chondromatosis of the finger.

Case No.	Age, Gender	Side, Finger	Surface	Tendon*	Nodule	Size (Area)	Cal/Oss	Intra-oss	OA	Duration	Follow-up
1	65, F	R, Index	Dorsal	Under	Multi	1 cm (x 5 cm)	+/–	+	–	40 y	2 y + 0 mo
2	46, F	R, Middle	Volar	Above	Mono	1.5 cm	–/+	–	–	6 mo	11 mo
3	66, M	R, Ring	Dorsal	Under	Multi	2 cm (x 2 cm)	–/–	–	–	3 mo	5 y + 2 mo

Cal: calcification, F: female, Intraoss: intraosseous lesion, L: left, M: male, mo: month, OA: osteoarthritic degenerative change in the nearby joint, Oss: ossification, R: right, Tendon*: relationship to the tendon, y: year.

## LIST OF ABBREVIATIONS

MP: = Metacarpophalangeal Joint
MRI: = Magnetic Resonance Image
PIP: = Proximal Interphalangeal
